# Supplementary material for: Physiological Significance of the Heterogeneous Distribution of Zeaxanthin and Lutein in the Retina of the Human Eye
Source: Int J Mol Sci. 2023 Jun 27;24(13):10702. doi: 10.3390/ijms241310702 (PMC10342121; doi:10.3390/ijms241310702)
Supplement: Supplementary file 1 [file ijms-24-10702-s001.zip › ijms-2426145-supplementary.pdf]

Supplementary Figures

Physiological Significance of the Heterogeneous Distribution of Zeaxanthin and Lutein in the Retina of the Human Eye

Wojciech Grudzinski [1,*,†], Rafal Luchowski [1], Jan Ostrowski [2,†], Alicja Sęk [3], Maria Manuela Mendes Pinto [1], Renata Welc-Stanowska [4], Monika Zubik-Duda [1], Grzegorz Teresiński [5], Robert Rejdak [2] and Wieslaw I. Gruszecki [1,*]

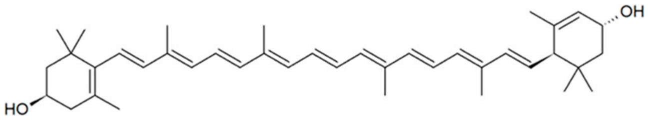

Lutein

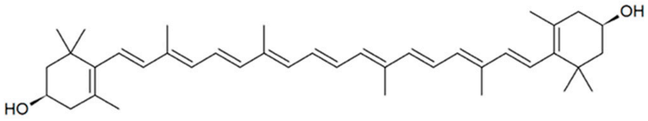

Zeaxanthin

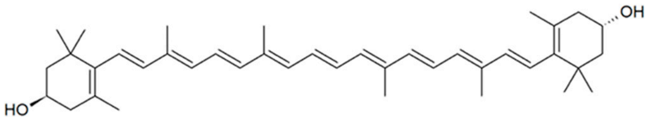

meso-Zeaxanthin

Figure S1. Chemical structures of the macular xanthophylls.

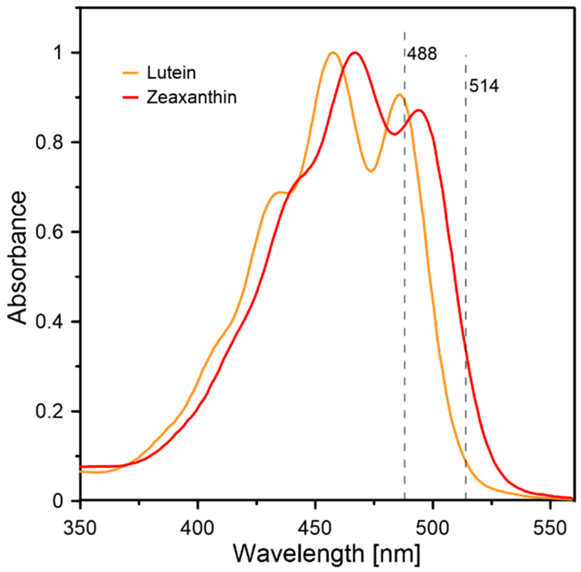

Figure S2. Absorption spectra of lutein and zeaxanthin dissolved in chloroform. The refractive index of the solvent selected (n=1.44) corresponds well to the dielectric properties of the hydrophobic core of lipid membranes. Absorption spectra recorded with Cary 50Bio UV-Vis spectrophotometer from Agilent Technologies.

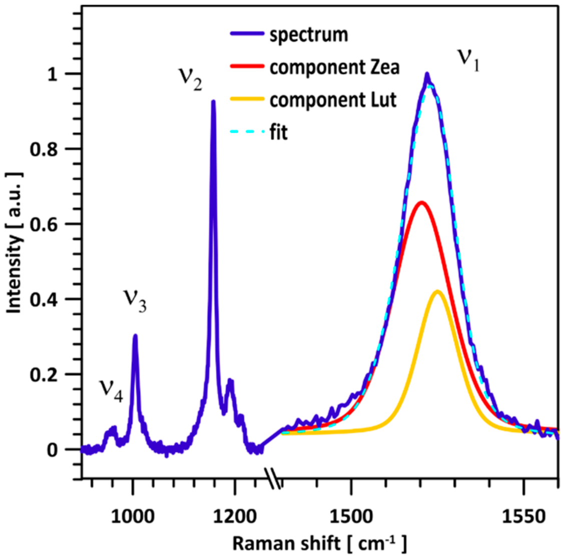

Figure S3. Raman spectrum recorded from the human retina with 514 nm laser along with the Gaussian deconvolution components. Gaussian deconvolution components of the ν_1 band, assigned to zeaxanthin (centered at 1521 cm^{-1}) and lutein (centered at 1526 cm^{-1}) are also shown along with the spectral fit. Characteristics of the spectral components assigned to lutein and zeaxanthin are based on the resonance Raman spectra of pure xanthophylls in lipid membranes [10]. Note that the ν_1 band representing the C=C stretching vibrations is shown on an enlarged scale. The retina from a healthy 34-year-old female donor.

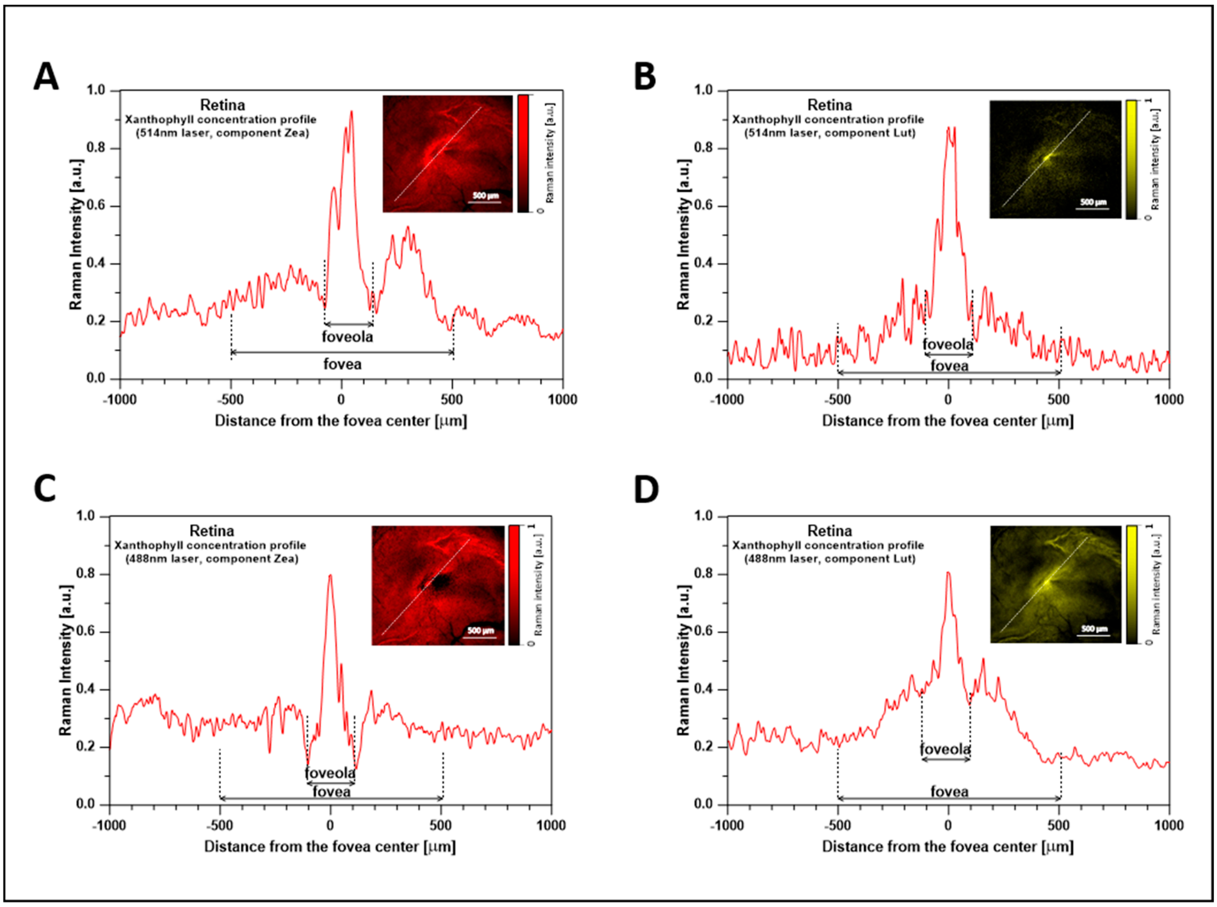

Figure S4. Optical cross-section analysis of the human retina. The analysis is based on the integration of the ν_1 band of Raman spectra recorded in the course of imaging the preparation shown in the inset (see Figure 2 of the article). The direction of the cross-section is drawn with the white dashed line. Panels represent the analysis based on the integration of the Gaussian components assigned to zeaxanthin (A, C) or lutein (B, D). The same retina fragment was scanned either with 514 nm (A, B) or with 488 nm (C, D) laser lines. The retina from a healthy 35-year-old male donor.

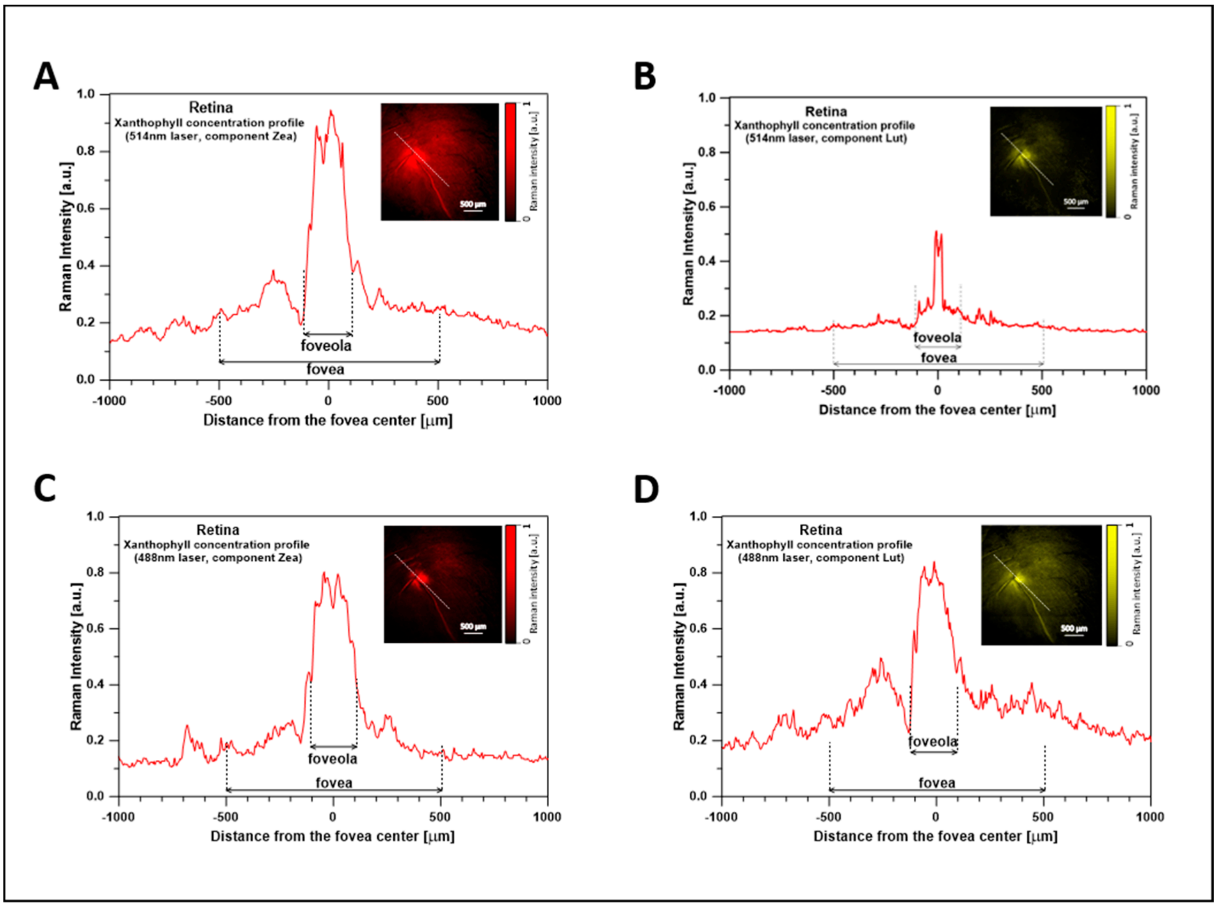

Figure S5. Optical cross-section analysis of the human retina. The analysis is based on the integration of the ν_1 band of Raman spectra recorded in the course of imaging the preparation shown in the inset (see Figure 3 of the article). The direction of the cross-section is drawn with the white dashed line. Panels represent the analysis based on the integration of the Gaussian components assigned to zeaxanthin (A, C) or lutein (B, D). The same retina fragment was scanned either with 514 nm (A, B) or with 488 nm (C, D) laser lines. The retina from a healthy 34-year-old female donor.

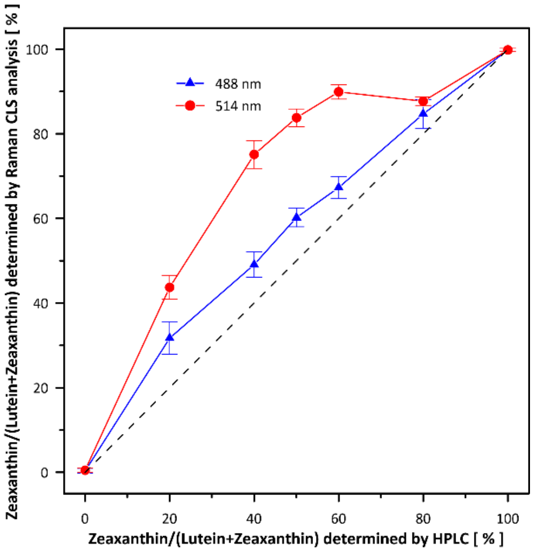

Figure S6: Validation dependencies of Raman spectroscopy-based determination of Lut and Zea fractions. Raman spectra were recorded from chromatographically pure 10-μM Lut and Zea mixtures in ethanol. Fractions of Lut and Zea were determined based on classical least square (CLS) spectral fitting of the spectra of pure components. Note the deviations from the ideal linear relationship (dashed line) depending on the current resonance conditions for Lut and Zea scanned with the 488 nm and 514 nm lasers. Experimental points are the average from 15 different determinations ± S.D.

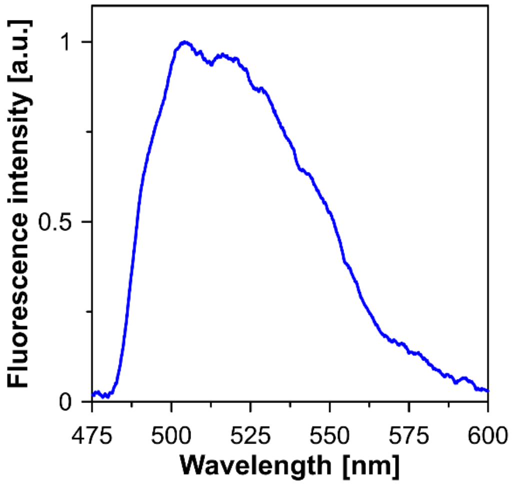

Figure S7. Fluorescence emission spectrum recorded from a single pixel in the image of a single axon (see Figure 6 of the article). The retina from a healthy 57-year-old male donor.
